# Combined Spraying of Boron and Zinc During Fruit Set and Premature Stage Improves Yield and Fruit Quality of European Hazelnut cv. Tonda di Giffoni

**DOI:** 10.3389/fpls.2021.661542

**Published:** 2021-05-31

**Authors:** Cristian Meriño-Gergichevich, Ana Luengo-Escobar, David Alarcón, Marjorie Reyes-Díaz, Gabrijel Ondrasek, Filis Morina, Khristopher Ogass

**Affiliations:** ^1^Departamento de Producción Agropecuaria, Facultad de Ciencias Agropecuarias y Forestales, Universidad de La Frontera, Temuco, Chile; ^2^Scientific and Technological Bioresource Nucleus (BIOREN-UFRO), Universidad de La Frontera, Temuco, Chile; ^3^Laboratory of Plant Physiology and Nutrition in Fruit Crops, Facultad de Ciencias Agropecuarias y Forestales, Universidad de La Frontera, Temuco, Chile; ^4^Laboratorio de Fisiología y Biología Molecular Vegetal, Departamento de Ciencias Agronómicas y Recursos Naturales, Facultad de Ciencias Agropecuarias y Forestales, Universidad de La Frontera, Temuco, Chile; ^5^Laboratory of Molecular and Functional Ecophysiology of Plants, Departamento de Ciencias Químicas y Recursos Naturales, Facultad de Ingeniería y Ciencias, Universidad de La Frontera, Temuco, Chile; ^6^Department of Soil Amelioration, Faculty of Agriculture, University of Zagreb, Zagreb, Croatia; ^7^Department of Plant Biophysics and Biochemistry, Institute of Plant Molecular Biology, Biology Centre of the Czech Academy of Sciences in Budweis, Ceske Budejovice, Czechia; ^8^Research and Extension Center for Irrigation and Agroclimatology (CITRA), Facultad de Ciencias Agrarias, Universidad de Talca, Talca, Chile

**Keywords:** nut traits, *Corylus avellana* (L.), foliar nutrition, kernel growth, radical scavenging activity

## Abstract

Boron (B) and zinc (Zn) are essential micronutrients of plant nutrition programs in orchards for securing the crop quality and yield. Although orchard supplementation with B and Zn is a common practice to overcome deficiencies or maintain their optimal levels, the efficiency of combined B and Zn spraying in relation to European hazelnut (*Corylus avellana* L.) phenological stage has not been investigated so far. Leaf and kernel mineral and functional traits were studied in cultivar Tonda di Giffoni after B and Zn spraying in four phenological stages. During the 2016/2017 season, 9-year-old trees were sprayed with B (0, 800, and 1,600 mg L^−1^) and Zn (0, 400, and 800 mg L^−1^) under three treatments: B_0_+Zn_0_, B_800_+Zn_400_, and B_1600_+Zn_800_ implemented in three spring application programs scheduled from October to December (P1: four times, P2: early two times, and P3: late two times). B and Zn treatments in P1 and P3 led to higher Zn concentration both in leaves and in kernels compared with non-sprayed trees. Stabilized nut production increased 2.5-fold under B_800_+Zn_400_ in all three programs. Kernel/nut ratio improved in both B+Zn treatments in P1 and P3, while the percentage of blank nuts was reduced compared with B_0_+Zn_0_. Increased radical scavenging activity in B+Zn-treated kernels and leaves was not attributed to the accumulation of phenolics in P3 compared with B_0_+Zn_0_, whereas B and Zn spraying reduced the level of lipid peroxidation in both studied organs. According to the results, combined B and Zn should be sprayed at the end of spring (P3) on hazelnut plantations in temperate areas such as Southern Chile, whereas early applications (P2) showed an irregularity in nut production and functional traits in nuts. Moderate and partialized rates of B and Zn and the time of implementation contribute to improving the quantitative and qualitative features crucial for future sustainable hazelnut production.

## Introduction

During the last years, European hazelnut (*Corylus avellana* L.) plantations have developed significantly in Chile, reaching 24,000 ha of planted surface in the 2018/2019 season (ODEPA-CIREN, 2019). World hazelnut production for 2019/2020 was 528,068 metric tons kernel basis, and after Turkey, Italy, Azerbaijan, Georgia, and the United States, Chile has taken an important place, as the first producer of hazelnut (35,000 t with shell) in the Southern Hemisphere (3% of world production), and it is the key off-season supplier of the Northern Hemisphere markets of both fresh and processed fruits (FAOSTAT, 2019; Nuts Dried Fruits Statistical Yearbook, 2020, Frutícola Agrichile S.A.). European hazelnut is an important source of antioxidants, such as phenolics, minerals, unsaturated fatty acids, vitamins, and other essential compounds, all beneficial for human health and diet (Blomhoff et al., 2006; Jakopic et al., 2011; Solar and Stampar, 2011; Bacchetta et al., 2013).

La Araucanía region in Southern Chile has over 7,000 ha of hazelnut plantations, and the commercial production covers both in-shell nuts and kernels (ODEPA-CIREN, 2019). However, in this region, hazelnut orchards are grown mainly on acidic soils (Andisols) derived from volcanic ashes in which chemical and biological properties such as low pH (≤5.5) and high organic matter impose difficulties for the adaptation of this crop. Moreover, acidifying fertilizers management and excessive liming can cause a reduced bioavailability of some micronutrients (Shorrocks, 1997; Marschner, 2012). Indeed, low B (0.5–1.0 mg kg^−1^) and Zn (<1.0 mg kg^−1^) concentrations in Andisols have been widely reported by Rodríguez and Tomic (1984), Rodríguez et al. (2001), and Bonomelli et al. (2003). Boron and Zn deficiencies are undoubtedly becoming important limiting factors for normal growth and development of fruit crops (Davarpanah et al., 2016; Meriño-Gergichevich et al., 2016) affecting metabolic pathways and resulting in reduced shoot growth, fruit set, and quality, as well as mineral and nutritional status of fruits (Özenç and Bender Özenç, 2015; Davarpanah et al., 2016; Marschner 2012). In this context, for hazelnuts planted in Southern Chile, leaf chemical analyses of the Laboratory of Soil and Plants and Scientific and Technological Bioresource Nucleus BIOREN-UFRO (www.bioren.ufro) at the Universidad de La Frontera have reported among 16–63 and 26–41 mg kg^−1^ DW (dry weight) of hazelnut cultivar Tonda di Giffoni (TDG) for B and Zn concentrations, respectively. According to technical extension reports, one approach for improving hazelnut nutrition, fruit set viability, and fruit filling, and for ensuring the production performance, is to maintain optimal B and Zn concentrations in plant tissues (Silva et al., 2003; Guerrero et al., 2015; Silvestri et al., 2021). In addition, Baron and Stebbins (1981), Ferrán et al. (1997), and Silva et al. (2003) recommended that spring B applications should be performed if hazelnut foliar analysis shows a B concentration below 100 mg kg^−1^ DW. Silvestri et al. (2021) reviewed a wide range of Zn concentrations (20–90 mg kg^−1^ DW) in adult leaves of different hazelnut cultivars such as Tonda Gentile, Barcelona, Tonda Romana, and Nocchione, indicating a cultivar-specific Zn accumulation; however, it is highlighted that the information about optimal Zn in tissues is scarcely studied for this species, particularly in Southern Chile. The beneficial effects of combined B and Zn spraying on vegetative and reproductive growth, yield, fruit traits, and nutrient mobility have been reported for apple (*Malus x domestica*) (Neilsen et al., 2004), olive (*Olea europaea*) (Saadati et al., 2013), walnut (*Juglans regia*) (Keshavarz et al., 2011), almond (*Prunus amygdalus*) (Nyomora et al., 1997), and in a lesser extent by studies conducted on hazelnut (Shrestha et al., 1987; Solar and Stampar, 2000; Alidust et al., 2020). In addition, foliar spraying is a convenient supplementation to traditional fertilization, demonstrating good effectiveness at low rates, uniformity of application, and very quick plant response, avoiding groundwater contamination, among other benefits (Fernández and Brown, 2013).

Both B and Zn contribute to the content of antioxidant compounds, phenolic acids, total oil, and protein content, as well as their amounts in kernels (Ozdemir and Akinci, 2004; Jakopic et al., 2011; Özenç and Bender Özenç, 2015). However, the effects of B and Zn spraying during fruit set or premature stage on the fruit antioxidants and redox homeostasis in hazelnut are scarcely studied. Reports on different fruit species are still controversial; e.g., Davarpanah et al. (2016) reported minor changes in total phenolic compounds and unaffected antioxidant activity in pomegranate (*Punica granatum* cv. Ardestani) sprayed in preharvest with B and Zn, while Saadati et al. (2013) reported an increased phenolic content after foliar application of B and Zn on olive during two productive seasons. Moreover, the effect of B and Zn concentrations or the frequency of application on oxidative stress determined as the level of lipid peroxidation (LP) in hazelnut tissues is not well-documented. Indeed, lipid oxidation negatively affects the quality and nutritional traits of nuts during storage, limiting the commercialization of hazelnut for processed food (Alasalvar et al., 2003).

Foliar applications of B and Zn have become routine in the annual nutrition program of commercial orchards in modern hazelnut production, with several administrations during the growing season, often combined with other compatible agrochemicals with the purpose to increase yields by improving fruit set and retention, reducing cluster and fruit drops, blanking or empty nuts, and enhancing kernel yield (Ferrán et al., 1997; Milošević and Milošević, 2012; Özenç and Bender Özenç, 2015). However, there is little knowledge concerning B and Zn interactions with hazelnut potential production as functional food in the coming years, to improve commercial opportunities for growers in Southern Chile and other productive areas worldwide with similar temperate weather and soil conditions. Thus, in this study, we investigated the effects of two combined B and Zn concentrations, applied in three spraying programs from early fruit set to initial premature stages on hazelnut leaves and fruit with the aim to obtain optimal nutritional and biochemical nut traits in hazelnut as a major commercial fruit in Chile.

## Materials and Methods

### Plant Material and Field Conditions

**Figure 1:**
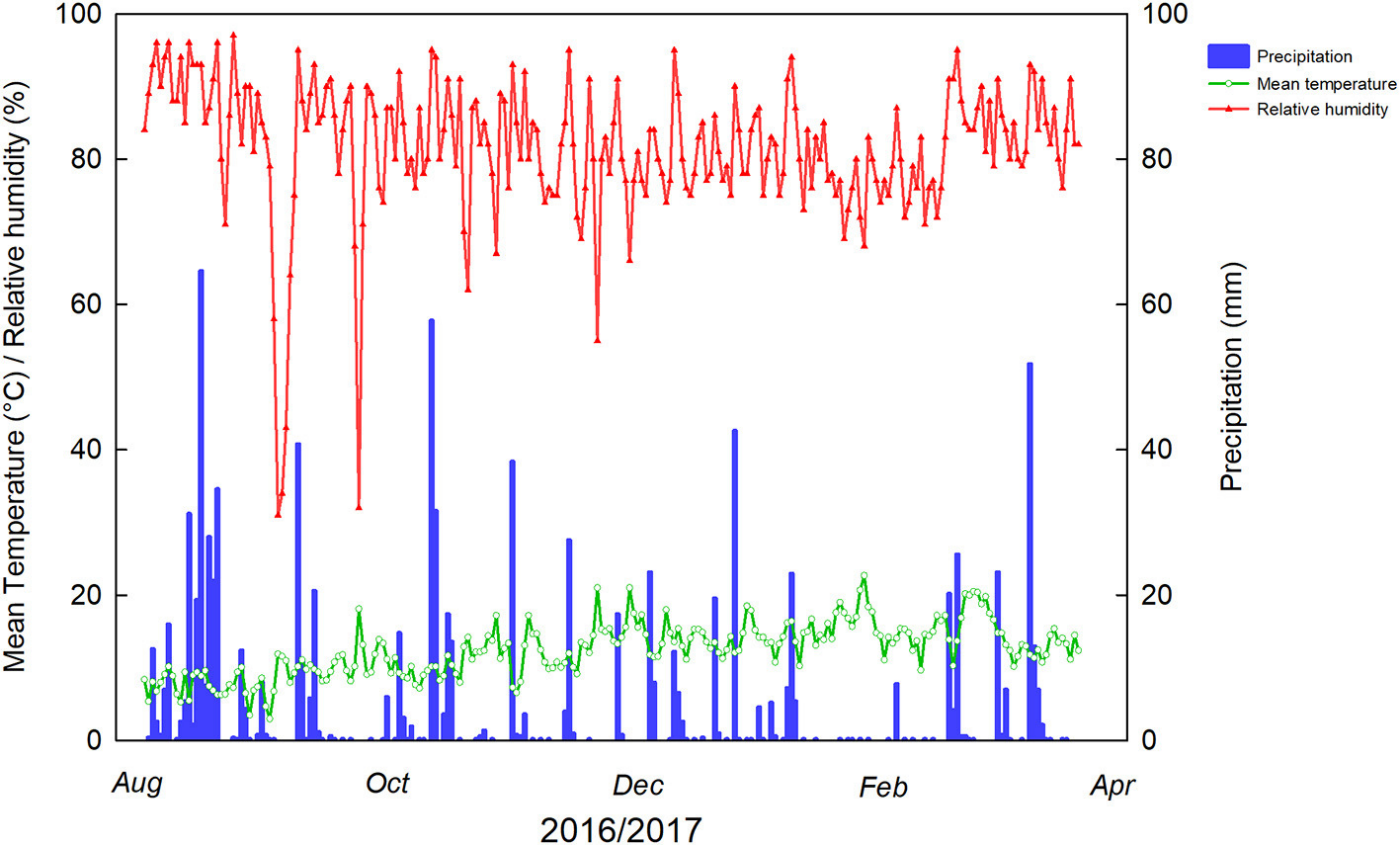
Weather parameters during the hazelnut vegetative season at the experimental site, Caracas Farm, Cunco (39° 00′ S, 72° 31′W), La Araucanía region, Chile. Minimum and maximum temperatures were −4.5°C (9/1/2016) and 34.6°C (1/26/17) during the studied period.

Experiments were conducted during the 2016/2017 (August 2016–April 2017) season in a commercial European hazelnut orchard in Caracas Farm (Frutícola Agrichile S.A.) located in Cunco (39° 00′S, 72° 31′W), La Araucanía region, Chile. Nine-year-old tree cultivar TDG were planted in regular rows with 5 ×4 m frame (500 trees ha^−1^) in a multistem system. This cultivar is one of the most planted in Southern Chile, covering 45% of the total hazelnut orchards surface and 55% of Chilean total production (Agrichile, 2020; Ascari et al., 2020). Soil orchard was an Andisol belonging to Freire Series (Typic Placudands), with flat topography and good drainage (CIREN, 2002) (Table 1). From August 2016 to March 2017, the mean temperature was 12.4°C, whereas the minimal and maximal average temperatures in the studied area were 6 and 20°C, respectively, with 946 mm of annual mean precipitation (data collected from the weather station in the experimental orchard <1 km) (Figure 1). Commercial agronomic management consisted of standard fertilization ranging from 90 to 110 N, 30 to 40 P, and 50 to 80 K (kg ha^−1^). The hazelnut orchard was weekly irrigated from November to March based on ETc, and water was supplied by drip irrigation system with a drip line irrigating each tree with emitters supplying water at a rate of 2.0 L h^−1^ spaced at 1.0 m. The water application was 4,000 m^3^ ha^−1^ during the studied period. Weed and basal shoots control was done with mechanical and chemical methods 3–4 times a year. Pest and disease management was done with 2–3 and 3–4 applications per year, respectively.

**Table 1:** Chemical properties of soil (Typic Placudands, Freire Series) and European hazelnut leaves from the tested commercial hazelnut orchard (Fundo Caracas, Frutícola Agrichile S.A.) in Cunco, La Araucanía region, Chile.

**Chemical property**	**Soil**	**Leaf**
N (mg kg^−1^)	25 ± 2.31	2,600 ± 32
P (mg kg^−1^)	6 ± 0.42	113 ± 3
K (mg kg^−1^)	156 ± 10	1,246 ± 7
pH (H_2_O)	6.0 ± 0.45	–
pH (CaCl_2_)	5.13 ± 0.04	–
Soil organic matter (%)	23 ± 1.87	–
Na (mg kg^−1^)	9.2 ± 0.00	162 ± 19
Ca (mg kg^−1^)	1,492 ± 216	6,430 ± 12
Mg (mg kg^−1^)	248 ± 1.22	1,400 ± 1
Al (mg kg^−1^)	3.6 ± 0.00	100 ± 16
Al sat (%)	0.40 ± 0.01	–
CEC (cmol+ kg^−1^)	9.98 ± 1.12	–
Σ basis (cmol+ kg^−1^)	1,908 ± 227	–
B (mg kg^−1^)	0.36 ± 0.01	25 ± 1
Zn (mg kg^−1^)	1.08 ± 0.04	29 ± 2
Cu (mg kg^−1^)	4.15 ± 0.90	–
Fe (mg kg^−1^)	33 ± 1.11	–
Mn (mg kg^−1^)	2.13 ± 0.04	–
S (mg kg^−1^)	26 ± 2.04	–
Ext. Al (mg kg^−1^)	1,100 ± 34	–

*Soil samples were collected from 0 to 30 cm depth in October 2016. Values represent the mean (n = 3) ± SE.*

† *Calculated as Al/cation exchange capacity [Σ (K, Ca, Mg, Na, and Al)] ×100*.

### B and Zn Spraying Arrangement

Experiments consisted of three combinations of B and Zn, with final concentrations of B (0, 800, and 1,600 mg L^−1^) and Zn (0, 400, and 800 mg L^−1^) that were sprayed on 12 trees per treatment and labeled as B_0_+Zn_0_ (control: only water), B_800_+Zn_400_, and B_1600_+Zn_800_, respectively (Table 2). These concentrations of B+Zn were applied in three programs (P1, P2, and P3) at four phenological stages during hazelnut vegetative growth in the Austral Hemisphere: P1, four spray applications: at the beginning of ovary development (October 15), fertilization stage (October 31), ovary growth (November 15), and nut growth/premature stage (November 30); P2, two spray applications: during ovary development (October 15) and in the fertilization stage (October 31); and P3, two spray applications: during ovary growth (November 15) and in the nut growth/premature stage (November 30). Boron and Zn were applied in the widely used form of Bortrac™ 150 (a.i 15% sodium perborate, Na_3_BO_3_ × H_2_O p/v, Yara United Kingdom Ltd., England) and Nutrizinc Plus (a.i 16% ZnCl_2_ p/v, Cytosine Laboratories Inc., United States), and sprayed in an output of 1,500 L ha^−1^ of water, determined by canopy volume (4πab^2^, a: ½ height; b: ½ width). Spraying was carried out using a back sprayer of 17 L (Cifarelli 1200, Cifarelli S.p.A-Voghera, Italia) with an output of 5 L min^−1^. The B and Zn concentrations in the water used for spraying (and for control B_0_+Zn_0_) were 0.012 and 0.081 mg L^−1^, respectively (standard methods for the examination of water and wastewater, APHA.AWWA.WEF. 22nd edition 2012).

**Table 2:** Experimental arrangement of spraying program, B and Zn concentrations (mg L^−1^) and time during season 2016/17 (austral hemisphere) in European hazelnut cultivar TDG (Fundo Caracas, Frutícola Agrichile S.A.), Cunco, La Araucanía region, Chile.

**Spraying program**	**B**	**Zn**	**Spraying date (day, month)**	
	**(mg L**^**−1**^**)**		**Oct**	**Nov**
Program 1 (P1)	0	0	15 and 31	15 and 30
	800	400		
	1,600	800		
Program 2 (P2)	0	0	15 and 31	–
	800	400		
	1,600	800		
Program 3 (P3)	0	0	–	15 and 30
	800	400		
	1,600	800		

*October 15-ovary development, October 31-fertilization stage, November 15-ovary growth, November 30-nut growth/premature stage*.

### Leaves and Nut Harvest

Thirty fully developed and healthy leaves were collected from each tree (four trees per replicate) 15 days after the last application within each spraying program and transported to the laboratory in polyethylene bags at 4°C in a portable cooler. One half was used for chemical analyses, and the other half was stored at −80°C for biochemical analyses. In the middle of March 2017 (average temperature of 14.7 ± 0.31°C and relative humidity of 84.2 ± 1.04%), nuts were harvested from the four trees in each replicate and immediately placed in a forced air oven (40°C) for 72 h to stabilize the fruit at 6% humidity for the quantification of the nut yield components. Thirty in-shell nuts randomly sampled from each tree (120 nuts per replicate) were measured for their length, width, and thickness using a caliper (digital caliper CALDI-6MP, Truper, Mexico). Nut and kernel widths were measured in two dimensions meaning width 1 (W1) and width 2 (W2) similarly as reported by Solar and Stampar (2011). The nuts were manually cracked, and the shell thickness was measured on the convex side of each half using calipers. Stabilized nuts and kernels were weighed on a precision balance (Model BA2204B, Biobase Meihua Trading, China) and packed in porous bags and stored in-shell at room temperature (18°C) until chemical analyses.

### Chemical Analysis

Soil samples were collected from 0 to 30 cm depth and analyzed prior to the start of the experiment (September 30, 2016) (Table 1). The soil pH, Ca, Mg, K, Na, and B were analyzed according to the protocol by Sadzawka et al. (2004). Total Zn was determined by the extraction method of Lindsay and Norvell (1978).

Prior to the start of the experiment (October 2016), the nutrient concentrations in fully expanded mature leaves and collected stabilized nuts were determined according to Sadzawka et al. (2004) (Table 1). Leaves were dried in a forced air oven (Memmert model 410, Schwabach, Germany) for 48 h at 70°C until a constant DW was reached. Samples were weighed and ashed for 8 h at 500°C (JSMF-30 T, electric muffle furnace of JSR Research Inc., Korea). Later, the ashes were digested with 2 M hydrochloric acid and filtered. All samples were measured by a simultaneous multielement atomic absorption spectrophotometer (model UNICAM 969 Atomic absorption Spectrometer, England, United Kingdom). All analyses were conducted in triplicate.

### Lipid Peroxidation, Total Phenolics Content, and Radical Scavenging Activity

As an oxidative stress indicator, LP was determined in fresh leaves and kernels using thiobarbituric acid reacting substance (TBARS) assay, according to the modified protocol of Du and Bramlage (1992). Absorbance was measured at 532, 600, and 440 nm to correct the interference generated by TBARS–sugar complexes. The results were expressed as nanomole of equivalents of malondialdehyde (MDA) contents per fresh weight (nmol MDA g^−1^ FW), a secondary product of the polyunsaturated fatty acid oxidation. Total phenolics content (TPC) was determined in the leaves and kernels following the method of Slinkard and Singleton (1997) using the Folin-Ciocalteu reagent. Hundred and fifty microliters of ethanolic extracts (80% v/v) was mixed with 450 μL of H_2_O and 100 μL of the Folin-Ciocalteu reagent. After 5 min, 300 μL of Na_2_CO_3_ (7% w/v) was added. The absorbance of the mixture was measured by a UV-VIS spectrophotometer at 765 nm (UV–Vis spectrophotometer SP 8001, Metertech Inc. Taipei, Taiwan), and TPC was expressed as μg of chlorogenic acid equivalents (CAE) per g^−1^ FW. For radical scavenging activity (RSA), 2,2-diphenyl-1-picrylhydrazyl (DPPH) assay was used; leaf and kernel samples (0.10 g) were homogenized using 1 ml of methanol (80% v/v) and centrifuged for 5 min at 10,000 rpm (4°C). The supernatant was collected and stored at −80°C until analysis. The free RSA of the methanol extracts was measured as the decrease in absorbance at 517 nm using Trolox as a standard and expressed as milligram of Trolox equivalent per gram of fresh weight (mg TE g^−1^ FW) (Yu et al., 2002).

### Statistical Analysis

The experiments were performed as a factorial completely randomized block design with three spraying programs, three treatments, and three replicates (3 ×3 ×3). The data were statistically evaluated by a two-way ANOVA to assess the significance of the main factors (spraying program and B+Zn treatment) and the significance of their interactions. A Tukey *post-hoc* test was used to determine the significance of differences among the means with a significance level at *P* ≤ 0.05. The relationships among variables were examined using Pearson's correlation analysis at a significance level of *P* < 0.05. The resulting *P*-values were corrected using the false discovery rate (FDR) script displayed by the Rbio software (www.biometria.ufv.br). To reduce the dimensionality of data set, a principal component analysis (PCA) was performed to identify the variables that explained a higher proportion of the total variance. For this analysis, all data were averaged and normalized [log (10)] to minimize the effect of different units of measurement in the variance of each component. The software XLSTAT-2021.1.1 was used for PCA (Addinsoft, 2021).

## Results

### Weather Conditions and Nutrient Concentration

Weather conditions of hazelnut orchard at the time of the experiments are given in Figure 1. The highest mean temperature during the spraying time was 21°C (November 21) concomitantly with 55% relative humidity, appropriate for foliar spraying, whereas in the preharvest time, 22°C was the highest temperature registered (January 26).

**Figure 2:**
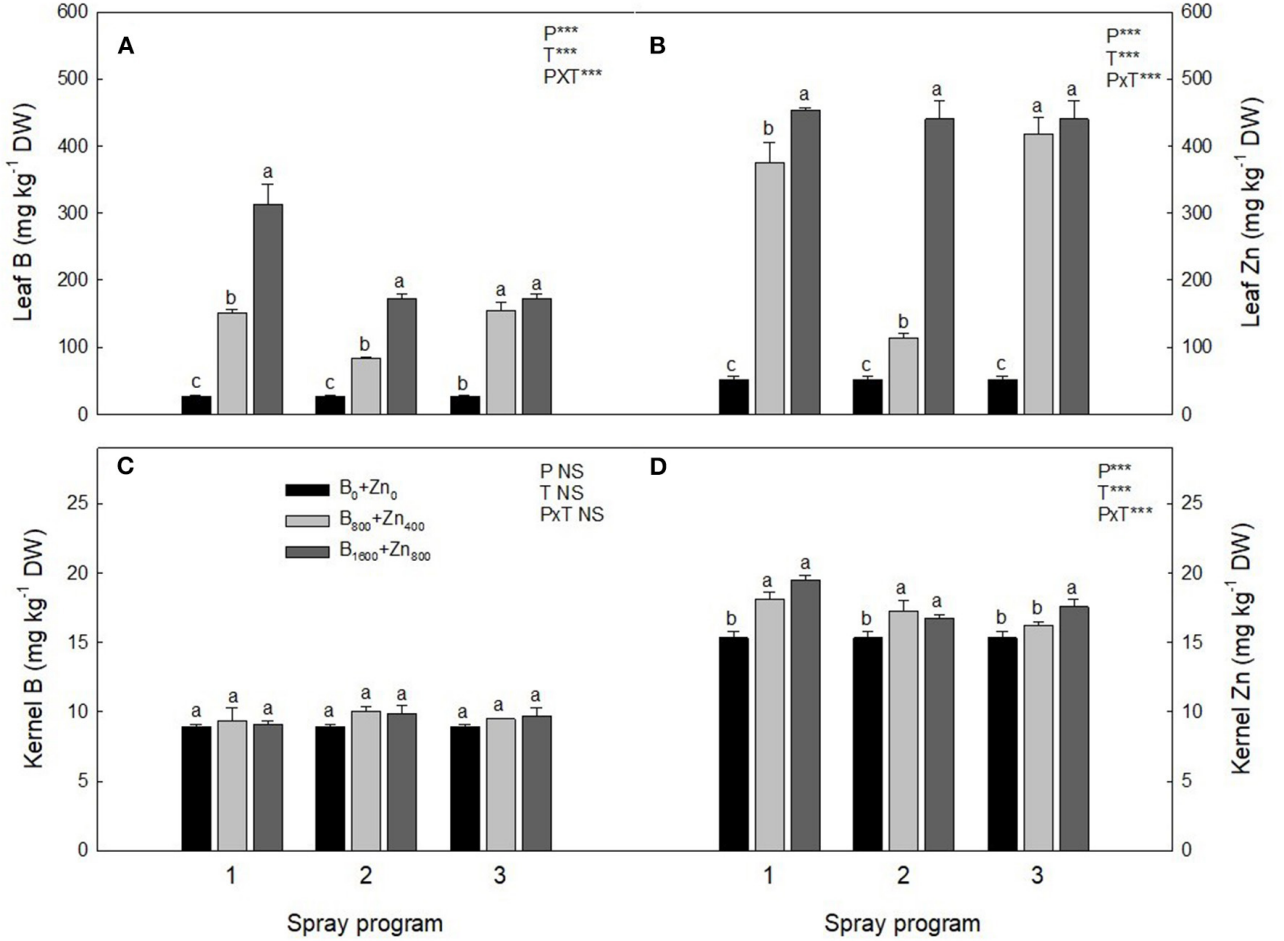
B and Zn concentration (mg kg^−1^ DW) in leaves **(A,B)** and kernels **(C,D)** of hazelnut trees depending on the applied spraying program. Values represent the mean (*n* = 3) ± SE. Different lowercase letters indicate statistically significant differences (*P* ≤ 0.05) among treatments within each spray program. Two-way ANOVA results are shown (NS, not significant, *P* > 0.05; ***P < 0.001).

Prior to the experiment, the concentrations of bioavailable B and Zn in the orchard soil were 0.36 and 1.08 mg kg^−1^, respectively, and soil pH_H2O_ was around 6.0 (Table 1). As expected, at the end of the experiments, leaf B and Zn concentrations in control trees (B_0_+Zn_0_) were similar as in the beginning, averaging 27 and 29 mg kg^−1^ DW for B and Zn, respectively. A significant interaction was found between spraying programs and B+Zn treatments (PxT, *P* < 0.001) for both leaf B and Zn concentrations; in P1- and P3-treated trees, B concentration was 90 and 87%, respectively, higher compared to B_0_+Zn_0_ (Figures 2A,B). However, the leaves of P2-treated trees had lower B and Zn concentrations than those of P1- and P3-treated trees for the same B and Zn treatments (B_800_+Zn_400_ and B_1600_+Zn_800_), yet still significantly higher than B_0_+Zn_0_ trees (*P* < 0.001). In the kernels, the overall B concentration ranged from 8.92 ± 0.14 to 10.05 ± 0.31 mg kg^−1^ DW, showing that neither B+Zn treatment nor spraying program had a significant effect on its accumulation (Figure 2C). Nevertheless, a significant interaction (PxT, *P* < 0.001) was found when Zn concentration was increased in stabilized kernels from 15.3 ± 0.49 (B_0_+Zn_0_) to 19.5 ± 0.29 mg kg^−1^ DW for B_800_+Zn_400_ and B_1600_+Zn_800_, respectively (Figure 2D), and a significantly higher amount of Zn in the kernels harvested from P1 trees was observed in B_1600_+Zn_800_ treatment compared to that from P2 and P3 trees.

**Figure 3:**
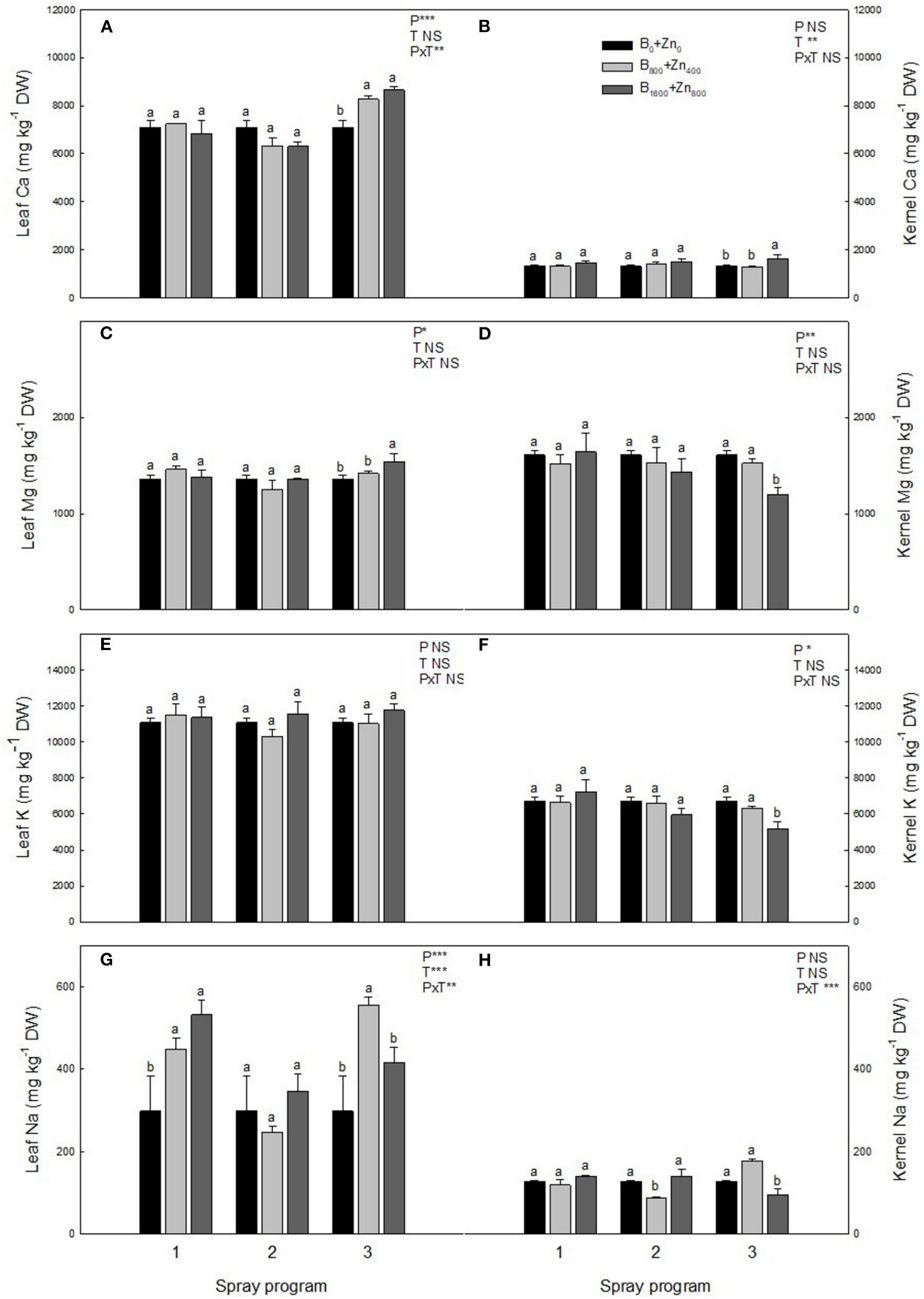
Calcium, Mg, K, and Na concentrations (mg kg^−1^ DW) in leaves **(A,C,E,G)** and kernels **(B,D,F,H)** of hazelnut trees depending on the applied spraying program. Values represent the mean (*n* = 3) ± SE. Different lowercase letters indicate statistically significant differences (*P* ≤ 0.05) among treatments within each spray program. Two-way ANOVA results are shown (NS, not significant, *P* > 0.05; *P < 0.05; **P < 0.01; ***P < 0.001).

In relation to other elements in the leaves, Ca and Na concentrations were influenced by the spraying program and B+Zn treatments (significant PxT interaction), showing a significant increase in Ca concentration of 14 and 18% in P3 B_800_+Zn_400_ and B_1600_+Zn_800_ treatments, respectively, in comparison with B_0_+Zn_0_ (*P* < 0.01; Figure 3A). Sodium concentration increased up to 44% in P1 and P3 B_800_+Zn_400_ and B_1600_+Zn_800_, but not in P2, in comparison with B_0_+Zn_0_ trees (*P* ≤ 0.01; Figure 3G). In addition, Mg concentration was higher in P3 B_1600_+Zn_800_ treatment compared with P1 and P2 (*P* < 0.05; Figure 3C) and compared with B_0_+Zn_0_-treated trees.

In the kernels, significantly increased Ca concentration in P3 B_1600_+Zn_800_ treatment compared with B_0_+Zn_0_ (Figure 3B) was found. Concomitantly, Mg concentration in the kernels was the lowest at B_1600_+Zn_800_ in P3 (*P* ≤ 0.05; Figure 3D), while no differences were observed for other programs/treatments. Despite the increase of Na in the leaves, in the kernels of P3 B_1600_+Zn_800_-treated trees, Na concentration decreased, while in P2, similarly as in the leaves, it decreased in the B_800_+Zn_400_ treatment compared with B_0_+Zn_0_ (significant PxT interaction, *P* ≤ 0.001) (Figure 3H). Boron and Zn treatments or programs did not affect K level in the leaves (Figure 3E), whereas K concentration decreased in the P2 and P3 B_1600_+Zn_800_-treated kernels by 24 and 28%, respectively, in comparison with P1 (*P* ≤ 0.05; Figure 3F).

### Nut Yield and Quality Features

**Figure 4:**
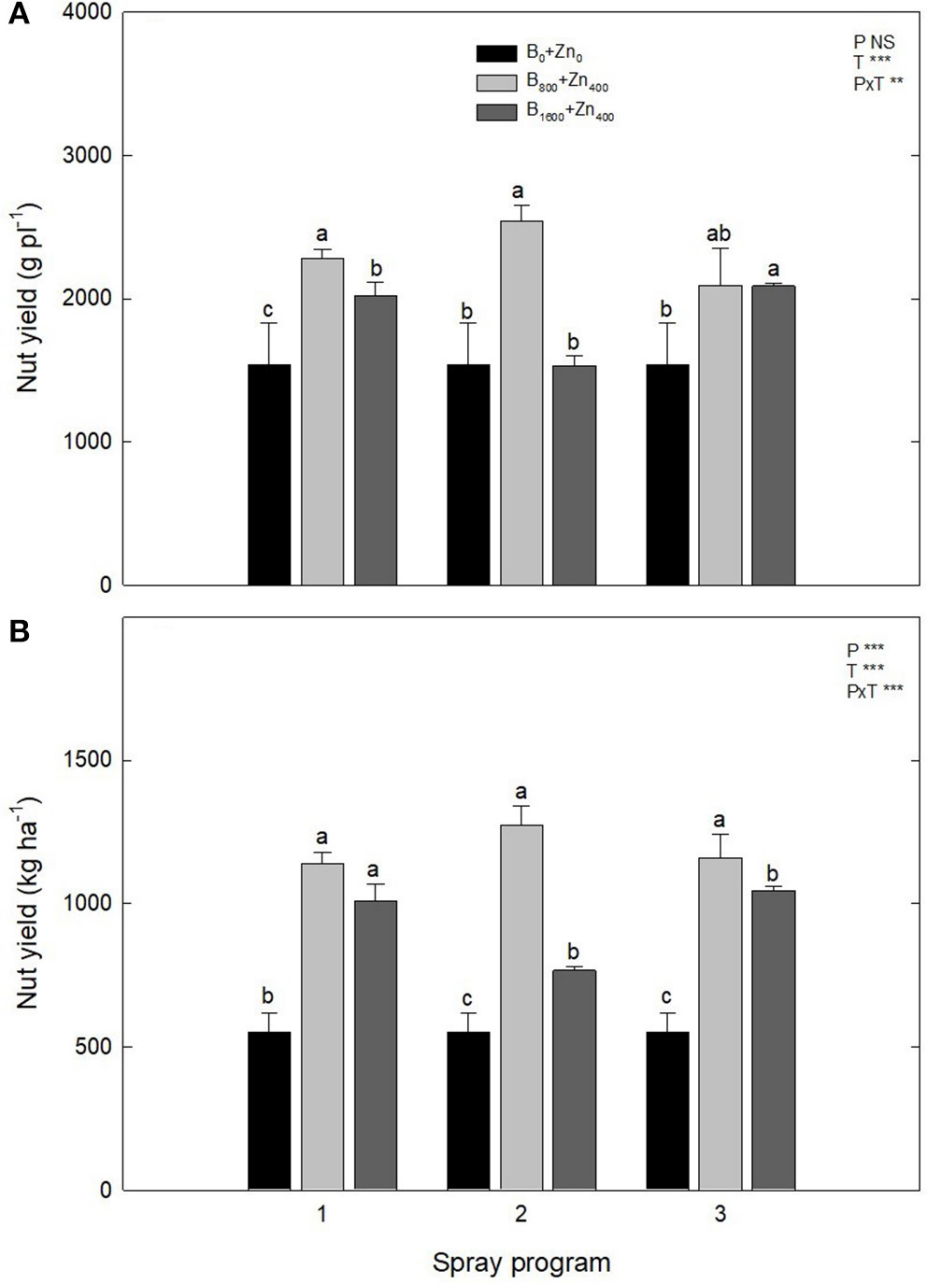
Effects of applied spraying programs on stabilized fruit yield per plant (g pl^−1^) **(A)** and per hectare (kg ha^−1^) **(B)**. Values represent the mean (*n* = 3) ± SE. Different lowercase letters indicate statistically significant differences (*P* ≤ 0.05) among treatments within each spray program. Two-way ANOVA results are shown (NS, not significant, *P* > 0.05; **P < 0.01; ***P < 0.001).

In all three spraying programs, B and Zn supplementation increased the in-shell nut yield, both per plant and per ha of orchard area in comparison with B_0_+Zn_0_ [significant PxT interaction for in-shell nut yield (g pl^−1^, *P* ≤ 0.01)] (Figure 4A). Conversely to what was expected by the farm technical staff, the trees subjected to B_800_+Zn_400_ showed higher productivity in all three spraying programs than those subjected to B_1600_+Zn_800_ treatment, especially in P1 and P2 (*P* ≤ 0.001). The orchard production of stabilized in-shell nuts was strongly augmented after B and Zn spraying, increasing from 550 ± 70 kg ha^−1^ in B_0_+Zn_0_ trees to ~1,273 kg ha^−1^ for B_800_+Zn_400_-treated trees in all three spraying programs (*P* ≤ 0.05), while no differences were observed between B_800_+Zn_400_ and B_1600_+Zn_800_ treatments in P1 and P3 (Figure 4B).

In stabilized nuts, industrial yield components such as weight (g), height (cm), and diameter (mm) were not significantly affected by B and Zn treatments and application program in comparison with untreated trees (Table 3). However, in the P1 B_1600_+Zn_800_ treatment, kernels diameter increased by 18% compared with B_0_+Zn_0_ (significant T × diameter interaction, *P* ≤ 0.05). Moreover, kernel yielding (%), one of the most important attributes of marketable nuts production, was improved in P1 and P3 B_1600_+Zn_800_ treatments, by 14 and 10% in comparison with B_0_+Zn_0_ (*P* ≤ 0.05). Moreover, the number of blank nuts was greatly reduced by B and Zn treatments in P1 and P3 (significant PxT interaction, *P* ≤ 0.05) (Table 4). The shell thickness and weight were not affected by B and Zn nor by the spraying program.

**Table 3:** Some nut and kernel industrial yield components in European hazelnut cultivar TDG planted in Cunco, La Araucanía region, Chile.

		**Nut**				**Kernel**				
		**Weight (g)**	**Height (mm)**	**Width (mm)**		**Weight (g)**	**Height (mm)**	**Width (mm)**		**Kernel (%)**
**Program**	**Treatment**			**1**	**2**			**1**	**2**	
1	B_0_+Zn_0_	2.53 ± 0.08a	18.44 ± 0.00a	18.75 ± 0.00a	16.77 ± 0.00a	1.03 ± 0.00a	12.40 ± 0.00a	12.45 ± 0.00b	10.25 ± 0.00a	41.54 ± 0.58ab
	B_800_+Zn_400_	2.37 ± 0.02a	19.05 ± 0.45a	19.96 ± 0.18a	16.49 ± 0.05a	1.02 ± 0.03a	12.70 ± 0.78a	13.42 ± 0.05ab	9.92 ± 0.07a	40.80 ± 1.90b
	B_1600_+Zn_800_	2.47 ± 0.15a	18.55 ± 0.16a	20.15 ± 0.60a	16.86 ± 0.68a	1.19 ± 0.04a	13.70 ± 0.21a	15.24 ± 0.27a	11.89 ± 0.44a	48.29 ± 1.49a
2	B_0_+Zn_0_	2.53 ± 0.08a	18.81 ± 0.55a	20.08 ± 0.67a	16.47 ± 0.56a	1.13 ± 0.06a	13.23 ± 0.64a	14.33 ± 0.49a	10.58 ± 0.25a	41.54 ± 0.58a
	B_800_+Zn_400_	2.64 ± 0.16a	18.53 ± 0.30a	20.18 ± 0.52a	16.89 ± 0.67a	1.08 ± 0.12a	12.79 ± 0.58a	14.09 ± 0.81a	10.73 ± 0.92a	41.37 ± 1.63a
	B_1600_+Zn_800_	2.52 ± 0.13a	19.46 ± 0.14a	19.67 ± 0.61a	16.89 ± 0.38a	1.17 ± 0.06a	13.69 ± 050a	14.80 ± 0.75a	11.45 ± 0.55a	45.61 ± 0.94a
3	B_0_+Zn_0_	2.53 ± 0.08a	18.53 ± 0.42a	20.24 ± 0.29a	17.53 ± 0.43a	1.28 ± 0.03a	12.94 ± 0.50a	14.49 ± 0.78a	11.13 ± 0.95a	41.54 ± 0.58b
	B_800_+Zn_400_	2.47 ± 0.20a	19.14 ± 0.27a	20.30 ± 0.77a	17.38 ± 0.55a	1.20 ± 0.05a	13.72 ± 0.58a	14.88 ± 0.25a	11.25 ± 0.15a	42.89 ± 1.11ab
	B_1600_+Zn_800_	2.36 ± 0.18a	18.83 ± 0.43a	19.63 ± 0.73a	16.64 ± 0.68a	1.11 ± 0.01a	13.75 ± 0.20a	14.91 ± 0.32a	11.38 ± 0.19a	45.88 ± 3.23a
P		NS	NS	NS	NS	NS	NS	NS	NS	NS
T		NS	NS	NS	NS	NS	NS	^*^	NS	^**^
PxT		NS	NS	NS	NS	NS	NS	NS	NS	NS

*Values represent the mean (n = 3) ± SE. Different letters indicate statistically significant differences (P ≤ 0.05) among treatments within each spray program. Two-way ANOVA results are shown (NS, not significant, P > 0.05;*

* *P < 0.05;*

** *P <0.01)*.

**Table 4:** Some nut shell properties after application of B and Zn spraying programs in hazelnut cultivar TDG planted in La Araucanía region.

**Program**	**Treatment**	**Shell thickness (mm)**	**Shell weight (g)**	**Blank nut (%)**
1	B_0_+Zn_0_	1.26 ± 0.00a	1.20 ± 0.00a	10.00a
	B_800_+Zn_400_	1.27 ± 0.03a	1.29 ± 0.04a	6.67b
	B_1600_+Zn_800_	1.35 ± 0.13a	1.28 ± 0.10a	0.01c
2	B_0_+Zn_0_	1.27 ± 0.08a	1.36 ± 0.13a	6.67a
	B_800_+Zn_400_	1.45 ± 0.06a	1.36 ± 0.09a	6.67a
	B_1600_+Zn_800_	1.41 ± 0.07a	1.33 ± 0.07a	10.00a
3	B_0_+Zn_0_	1.34 ± 0.06a	1.43 ± 0.09a	6.67a
	B_800_+Zn_400_	1.29 ± 0.05a	1.46 ± 0.11a	3.33b
	B_1600_+Zn_800_	1.44 ± 0.05a	1.29 ± 0.14a	0.00c
P		NS	NS	^**^
T		NS	NS	^***^
PxT		NS	NS	^*^

*Values represent the mean (n = 3) ± SE. Different letters indicate statistically significant differences (P ≤ 0.05) among treatments within each spray program. Two-way ANOVA results are shown (NS, not significant, P > 0.05;*

* *P < 0.05;*

** *P <0.01;*

*** *P <0.001)*.

### Variation in Antioxidant Properties Under Different B and Zn Spraying Programs

**Figure 5:**
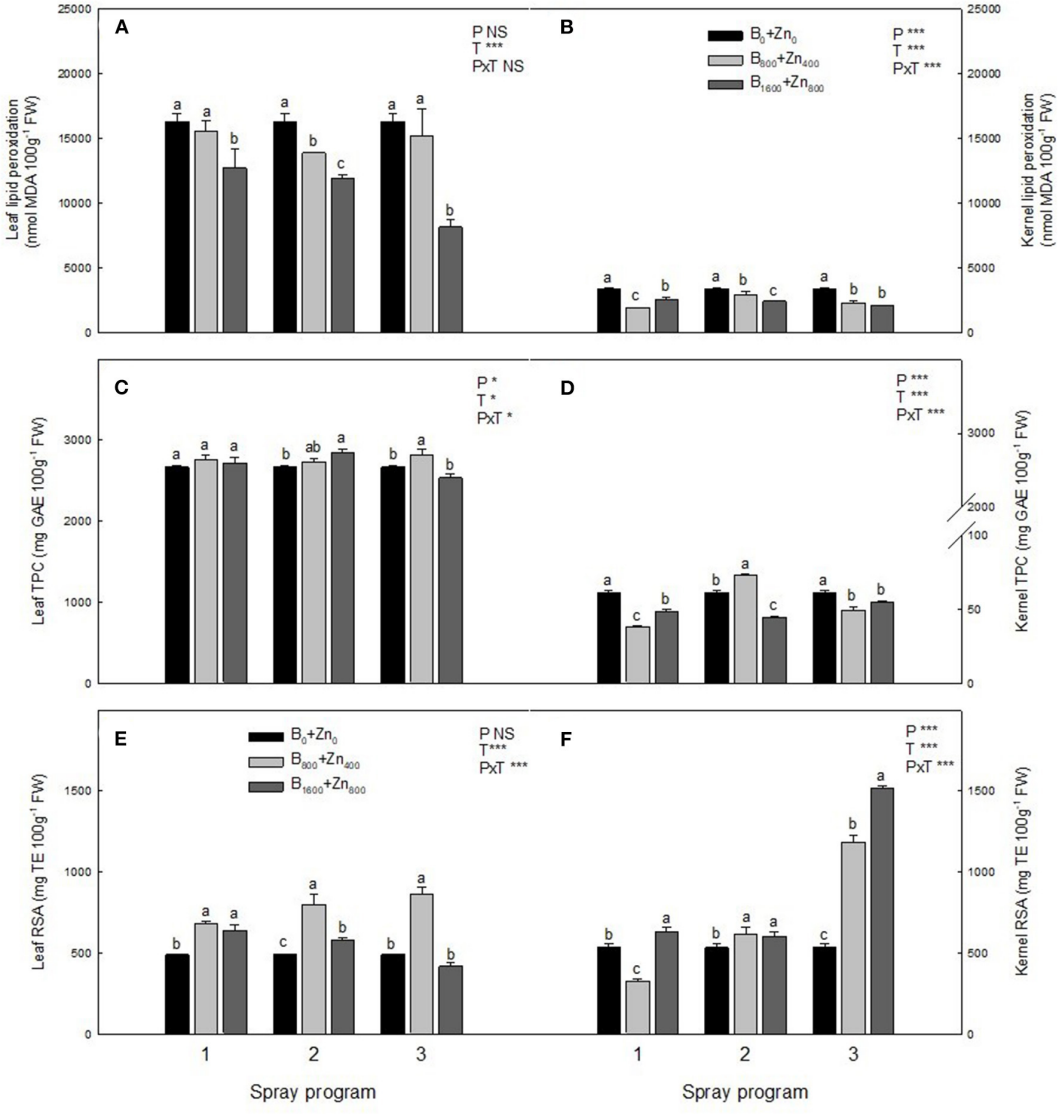
Effects of applied spraying programs on level of malondialdehyde (MDA), phenols content (TPC), and radical scavenging activity (RSA) in leaves **(A,C,E)** and kernels **(B,D,F)** of hazelnut cultivar TDG. Values represent the mean (*n* = 3) ± SE. Different lowercase letters indicate statistically significant differences (*P* ≤ 0.05) among treatments within each spray program. Two-way ANOVA results are shown (NS, not significant, *P* > 0.05; *P < 0.05; ***P < 0.001).

Boron and Zn treatments reduced the level of LP in both leaves and kernels in all three spraying programs (Figures 5A,B). In all programs, B_1600_+Zn_800_ drastically decreased LP levels in hazelnut leaves in comparison with B_0_+Zn_0_ (*P* ≤ 0.05). Kernel LP levels were lower than those observed on the leaves, and both B+Zn treatments reduced this oxidative stress marker by 43% in both tissues (significant PxT interaction, *P* ≤ 0.001). Leaf and kernel TPC and RSA are presented in Figures 5C–F, showing significant PxT interactions in both tissues. In the leaves, the highest RSA, determined by DPPH activity, was in B_800_+Zn_400_ treatments in all three programs. Particularly in the P3 kernels, RSA increased about 3-fold with both B_800_+Zn_400_ and B_1600_+Zn_800_ treatments compared with B_0_+Zn_0_ (significant PxT interaction, *P* ≤ 0.001).

### Pearson's Correlation Analysis

**Figure 6:**
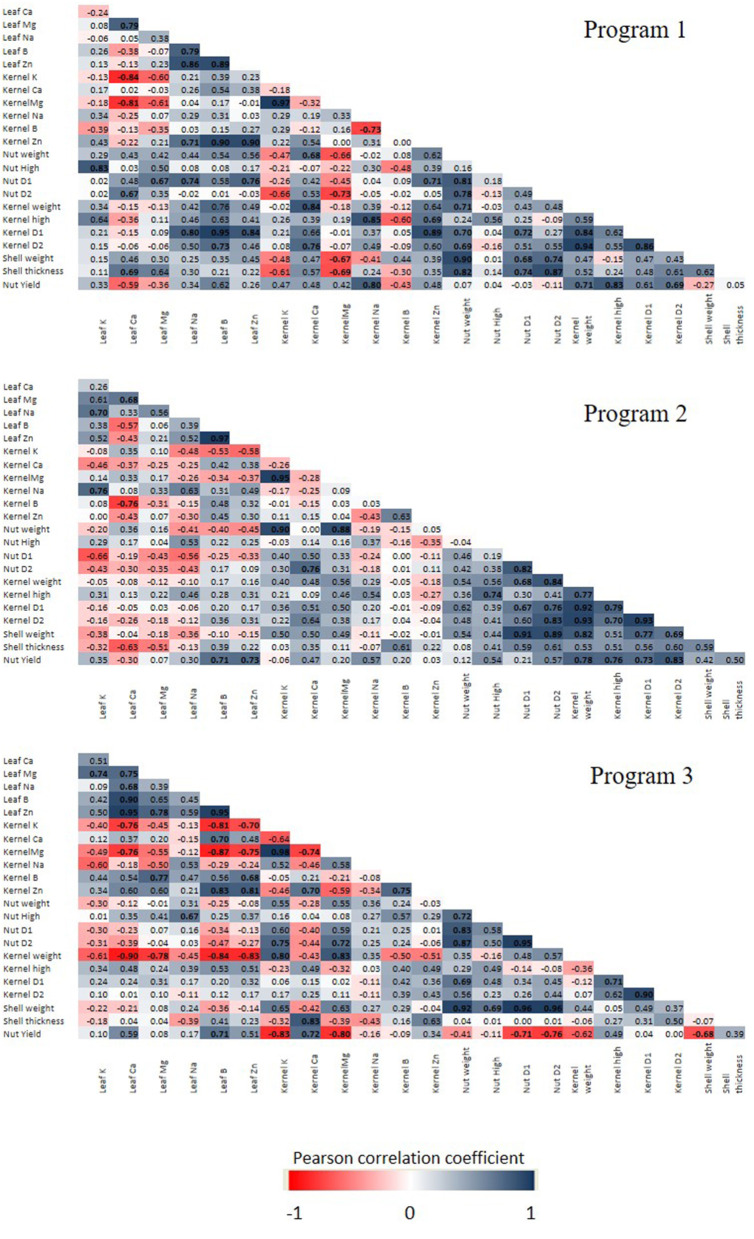
Pearson's correlation coefficient matrix between nutrient concentration and yield performance of hazelnut cultivar TDG. Correlation was calculated at three spraying programs (1, 2, and 3), and three B (0, 800, and 1,600 mg L^−1^) and Zn (0, 400, and 800 mg L^−1^) concentrations. Pearson's coefficient that is significant at *P* < 0.05 is indicated by boldface. Positive and negative correlations are distinguished by blue and red colors, respectively.

To determine the level of association among the measured variables for each program, Pearson's correlation matrices were performed for all pairs of the measured parameters related to nutrient concentration, yield, and nut quality features (Figure 6). At the 5% level of significance, a total of 131 correlations were found, out of which 108 were positive and 23 were negative. The P1 showed higher positive correlations than P2 and P3 (43), highlighting that leaf B concentration had a high correlation with kernels D1 and D2 and with Zn concentration in the kernel. Also, leaf B and Zn concentrations were strongly correlated with leaf Na concentration. Interestingly, the nut yield was not correlated with the levels of B and Zn in the leaf or kernel in this program, but only with the levels of Na in the kernels. A different trend was observed in P2, where 32 positive and only one negative correlation were found. The positive correlation of the B+Zn levels in the leaves with the nut yield is highlighted; however, the levels of B and Zn in the kernels were not related to other parameters except the negative B correlation with leaf Ca concentration. On the other hand, P3 had 33 positive and 16 negative correlations. Here, only leaf B and kernel Ca were positively correlated with nut yield, whereas leaf B and Zn were highly correlated with leaf Ca.

**Figure 7:**
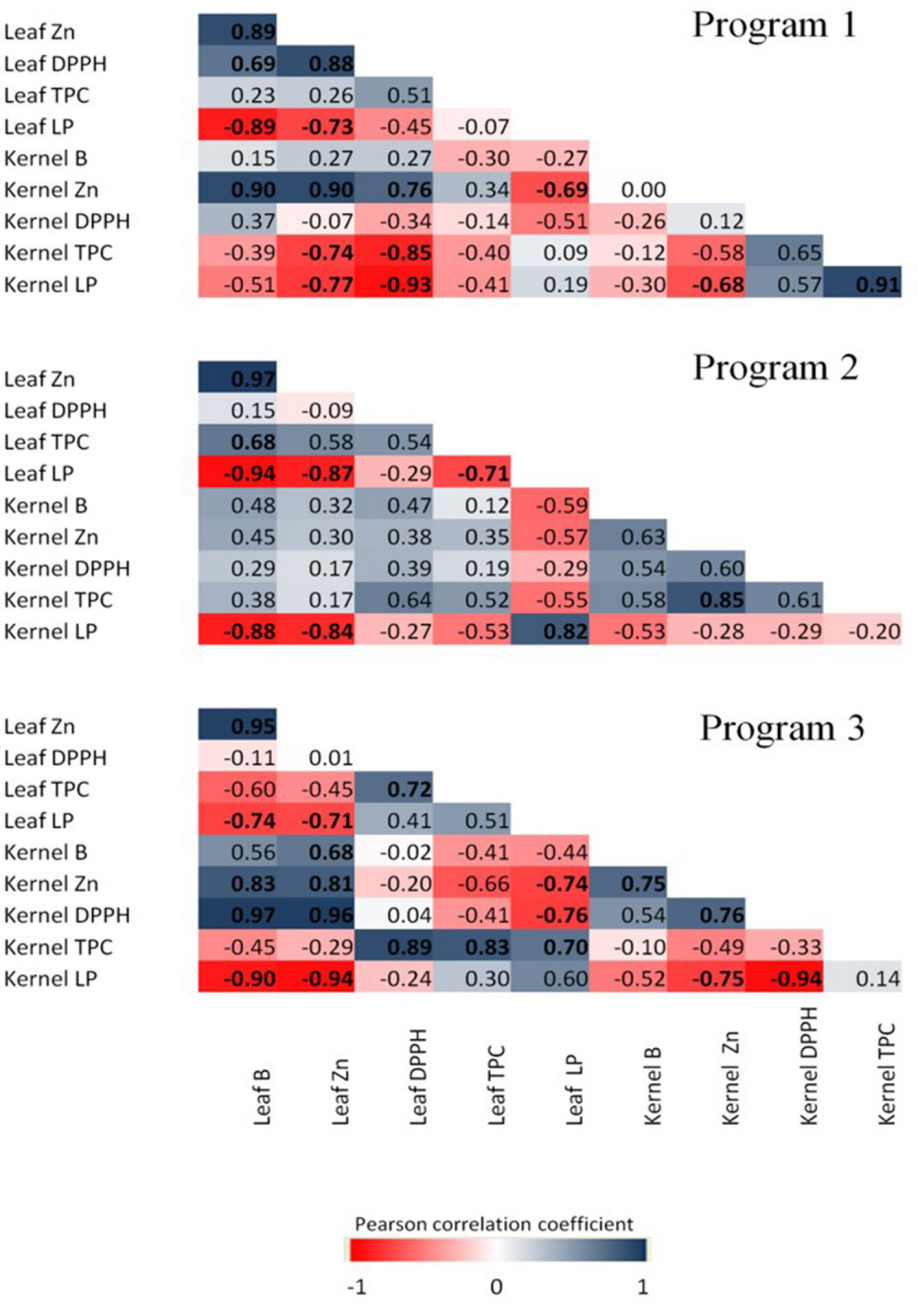
Pearson's correlation coefficient matrix among nutrient concentration and antioxidant features of cultivar TDG. Correlation was calculated at three spraying programs (1, 2, and 3), and three B (0, 800, and 1,600 mg L^−1^) and Zn (0, 400, and 800 mg L^−1^) concentrations. Pearson's coefficients that are significant at *P* < 0.05 are indicated by boldface. Positive and negative correlations are distinguished by blue and red colors, respectively.

Pearson's correlation coefficients between nutrient concentrations and oxidative and antioxidative parameters in the different programs were also analyzed. A total of 43 significant correlations were found, of which 22 were positive and 21 were negative. In P1, seven positive and eight negative significant correlations were found. Leaf B concentration was strongly correlated with the RSA capacity in the leaves, but not in the kernel. Leaf B concentration was highly correlated with kernel Zn concentration. Also, leaf RSA was negatively correlated with the level of LP in the leaf and kernel. In addition, LP was found to be negatively correlated with the levels of B and Zn in the leaves, while in the kernels, only Zn leaf and kernel levels were significantly and negatively correlated with kernel LP. In P2, three positive and five negative significant correlations were found. Leaf B had a strong positive correlation with TPC in the leaves, but not in the kernels, and a strong negative correlation with leaf and kernel LP, similar to the leaf Zn correlation with the same parameters (Figure 7). Levels of Zn in the kernels were strongly correlated with the levels of TPC in the kernels, while leaf TPC had negative correlation with leaf LP. In P3, a different trend was observed, and 12 positive and eight negative significant correlations were found. Leaf B was not correlated with RSA in the leaves. However, leaf B was negatively correlated with leaf and kernel LP. Leaf Zn was also negatively correlated with LP in the leaves and kernels, and highly correlated with kernel B, Zn, and antioxidant activity. However, the leaf antioxidant activity was highly correlated with TPC in leaves.

### Principal Component Analysis

**Figure 8:**
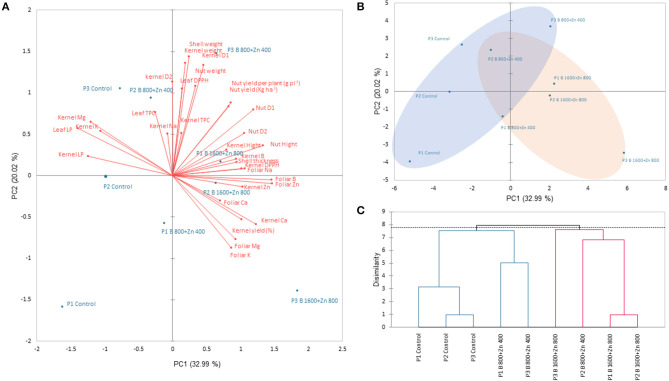
Principal component analysis (PCA) based on Pearson's correlation. Numbers in parentheses give the percent of the total variation explained by the PC1 and PC2, both representing 51.01% of the total variance. **(A)** Biplot of correlation, showing the treatments and variables together. **(B)** Principal component score plot. Treatments are clustered in two groups represented as blue and red ellipses. **(C)** Agglomerative hierarchical clustering (AHC) based on dissimilarity. The dotted line represents the automatic truncation, leading to two groups, which are represented in **(A)** by blue and red ellipses.

The first component (PC1) explained 32.99% of the total variance, and the most important variables were the concentration of B and Zn on leaves, leaf and kernel LP, kernel DPPH, Ca, Mg, B, and Zn, nut height, and width (D1 and D2). On the other hand, the PC2 explained 20.02% of the total variance; PC2 comprised the variables kernel weight, shell weight, kernel D1, D2, and nut weight as the most important (Figure 8A). In the PCA score plots, we observed a separation between programs and B and Zn treatments. Controls (B_0_+Zn_0_) of P1, P2, and P3 were clustered together with P1 and P3 for B_800_+Zn_400_ (Figure 8B, blue ellipse); meanwhile, the second cluster was composed of P2 B_800_+Zn_400_ and P1, P2, and P3 B_1600_+Zn_800_ (red ellipse). An agglomerative hierarchical clustering (AHC) was performed to find the clusters among treatments evaluated in this research (Figure 8C).

## Discussion

Agronomic practices in Chile have introduced both B and Zn nutritional programs in different orchards and emphasized that foliar sprays can compensate for nutrient deficiencies or maintain their plant levels following chemical soil and leaf analyses and orchard monitoring. Traditionally, companies have recommended guidelines for the application of micronutrients, particularly in fruit crops such as apple, blueberry, walnut, and hazelnut, as important commercial plantations in Chile. Indeed, nutritional strategies of B and Zn in hazelnut planted in Chile have been performed by growers to ensure their levels in leaves (31–75 mg B kg^−1^ DW and 16–60 mg Zn kg^−1^ DW) by soil and spray applications according to extension works from international experiences. Spraying of B and Zn has demonstrated an increased nut set even in non-deficient trees, when two or four sprayings are carried out, using concentrations to be sprayed from 500 to 2,000 mg L^−1^ or applied as fertilizer to the soil at 2–8 kg ha^−1^ rates. However, hazelnut grown in specific conditions of Southern Chile demands a comprehensive approach to nutritional management particularly of micronutrients.

### Effects of Different B and Zn Spraying Programs on Hazelnut Leaves

In our experiments with hazelnut TDG grown in acidic soil, the leaves of untreated, control trees had 25–28 mg kg^−1^ DW of B and Zn. According to technical reports, the optimal B leaf contents for reaching good fruit set and hazelnut yields are between 25 and 30 mg kg^−1^ DW (Silvestri et al., 2021). Similarly, other authors have discussed that optimum B concentration in hazelnut leaves should be 22–30 mg kg^−1^ DW for productive trees (Simşek et al., 2003), while data on the beneficial effect of Zn spraying on hazelnut leaves have been studied to a lesser extent (Alidust et al., 2020). Ferrán et al. (1997) reported even lower leaf B concentrations (14–21 mg kg^−1^ DW) to be optimal for hazelnut cultivars Pauetet and Negret planted in B-deficient soils (0.20–0.30 mg kg^−1^). We showed that the application of spraying programs (P1, P2, and P3) increased the B and Zn concentrations in the hazelnut leaves by several folds in both B+Zn treatments compared with untreated trees and the previous reports. It should be noted that the B and Zn requirements and optimum thresholds are cultivar-specific (Alidust et al., 2020; Silvestri et al., 2021), and they should be integrated into crop management to obtain the maximal yield. Our results showed that combined spraying application of B and Zn resulted in both Zn and B accumulation in totally expanded leaves in P1 and P3 programs, while the B and Zn accumulation efficiency was lower in P2 (*P* ≤ 0.05). The Zn concentration in sprayed TDG leaves was higher in comparison with the study conducted by Milošević and Milošević (2012) on hazelnut cultivars Tonda Gentile Romana, Nochione, and Istarski Duguljasti (12–14 years old) planted on the acidic Vertisol (pH 5.2) in Western Serbia. Indeed, Shear and Faust (1980) and Swietlik (2002) reported a wide range 10–200 mg kg^−1^ DW of normal Zn in the leaf of deciduous tree fruits and nuts.

Although B and Zn accumulation significantly increased in the leaves, in both treatments, lower LP levels and increased RSA compared with untreated trees were observed, thus excluding potential B and Zn toxic effects. Both B and Zn may modulate the profile of leaf metabolites and mitigate oxidative stress (Meriño-Gergichevich et al., 2016; García-López et al., 2019; Alidust et al., 2020). Landi et al. (2013) reported that B accumulation in the leaves of sweet basil (*Ocimum basilicum*) resulted in an increased RSA by increasing ascorbic acid, glutathione, and anthocyanin contents.

Boron and Zn supplementation may modulate the uptake and accumulation of other nutrients. For instance, Hosseini et al. (2007) reported synergistic B and Zn interactions on corn (*Zea mays*) growth and accumulation of other nutrients such as N, P, K, and Ca. In our experiments, the program type had an influence principally on Ca concentration in the leaves, while K levels were not affected by B and Zn supplied. However, PC1 showed that these variables are highly correlated, so the relation of these nutrients is evident principally in the leaves (Figure 8A). Exceptionally, Na accumulated in the leaves exposed to B and Zn treatments in P1 and P3 programs (PxT) was higher than expected, but it was not excessively high for the hazelnut leaves (246 ± 16.64–556.0 ± 18.4 mg kg DW) visible also from a decreased level of LP compared with untreated trees (Figure 5A). One possibility of increased Na level in B_800_+Zn_400_ and B_1600_+Zn_800_ under P1 was commercial fertilizer used as B source during the experiment (Na_3_BO_3_ × H_2_O). In our experiment, the collected kernel from all treatments showed lower Na (from 118 ± 13.8 to 139 ± 3.10 mg kg^−1^ DW) than reported by Ozdemir and Akinci (2004) for cultivars Palaz (379.5 ± 13.5 mg kg^−1^ DW), Tombul (508.5 ± 27.5 mg kg^−1^ DW), Cakildak (382.0 ± 7.0 mg kg^−1^ DW), and Karas (410.0 ± 3.0 mg kg^−1^ DW). However, Özenç and Bender Özenç (2015) reported no significant effect in cultivar Tombul after Zn fertilization (0, 0.2, 0.4, 0.8, and 1.6 kg ha^−1^) on Na in kernels ranging from 27.2 ± 0.55 to 30.6 ± 0.71 mg kg^−1^ DW. In fact, this variable was among the less influenced in PCA, in leaves and kernels (Figure 8A).

Interestingly, the leaves of trees in the P1 (four sprayings from ovary development until nut growth/premature stage) and P3 (two sprayings from ovary to growth; nut growth/premature stage) programs had similar B concentrations. These results suggest that delayed spraying (ovary and nut growth stages) and reduced B dosage (two sprayings) are sufficient for B enrichment of hazelnut leaves in comparison with current agronomic orchard management. Thereby, rational foliar fertilization has proved to be an important tool considering nutrient-deficient soils caused by natural or anthropogenic factors, and phenological stage of crops, with advantages such as quick plant responses, lower concentrations of added nutrients, uniformity, and less groundwater contamination (Keshavarz et al., 2011; Fernández and Brown, 2013; Zhang et al., 2016). Previous studies have emphasized the synergistic effect of combined B and Zn applications in several cropped species (Keshavarz et al., 2011; Mukhopadhyay and Mondal, 2015; Davarpanah et al., 2016; Alidust et al., 2020), particularly as supplementation program frequently recommended during springtime in fruit orchards (Solar and Stampar, 2000; Keshavarz et al., 2011; Meriño-Gergichevich et al., 2016).

### Effects of Different B and Zn Spraying Programs on Hazelnut Fruits

Beneficial effects of conventional B fertilization or spraying/foliar application on fruit set have been widely reported (Hanson and Breen, 1985; Erdogan and Aygun, 2009; Keshavarz et al., 2011; Alidust et al., 2020). However, the reports are often contradictory about B application methods for hazelnut production. For example, Kelley (1980) and Ferrán et al. (1997) reported no positive effects of B foliar application on hazelnut fruit set and yields. On the other hand, Erdogan and Aygun (2009) reported positive effects of B application on cultivar Tombul fruit set and concluded an annual dependency, although trees in ocak (training system commonly used in Turkey) were sprayed just one time with 300 and 600 ppm of B (H_3_BO_3_). Silva et al. (2003) concluded that weather conditions influenced the yields in hazelnut (cultivar Butler), more than B spraying, although nut mass and kernel mass were increased with B supplementation.

Zinc concentration in stabilized control kernels was lower (15.3 ± 0.49 mg Zn kg^−1^ DW) than reported by Özenç and Bender Özenç (2015) for cultivar Tombul without a Zn fertilization program (26.2 mg Zn kg^−1^ DW), possibly due to different soil properties and cultivar-specific Zn accumulation. While B readily accumulated in the leaves, its concentrations in the kernels were unchanged. Contrarily, Zn accumulated in the kernels in all three programs compared with untreated trees indicated differential mobility of the two nutrients from the leaf to the kernels. Zn mobilization from the leaves to grains has been reported previously (reviewed by White and Broadley, 2009). Nevertheless, the concentrations of B and Zn in treated kernels in our experiments showed referential dietary values of recommended daily amount (RDAs) being an adequate source of daily B and Zn intake similarly as shown by Özenç and Bender Özenç (2015). These authors have reported a mean kernel B concentration of 27.8 mg kg^−1^ DW in untreated trees (25 years old) of cultivar Tombul.

Hazelnut is not a high-yielding crop, and its maximum expected potential yield is only 4.5 t ha^−1^ (Mehlenbacher, 1991; Silvestri et al., 2021). In La Araucanía, the production of cultivar TDG is less than half of the maximal, as between 2014 and 2019 the mean production was 1.89 t ha^−1^, 32% less than in the orchards planted in Chilean Mediterranean ecosystems such as El Maule, with average productions of 2.8 t ha^−1^. Our previous studies within research projects Fondecyt 11160762 (2016/2019) and Corfo 16PTECFS-66647 (2017–to date) have determined the production ranges of 2.0 t ha^−1^ for cultivars TDG and Barcelona planted in La Araucanía when orchards (7–11 years old) were subjected to sustainable nutrition strategies that included macro- and micro-nutrient application management to get an attainable yield.

The beneficial effects of B and Zn spraying and their interactions on plant growth, fruit size, and higher uptake of macro- and micronutrients and oil content have been reported for nut species, such as walnut (*Juglans regia* L.) and hazelnut (Serdar et al., 2005; Alidust et al., 2020). On the other hand, in northern Portugal, Silva et al. (2003) did not find any significant differences in fruit set and yield in hazelnut B-sprayed (0, 300, 600, and 900 mg L^−1^) four times after blooming. Often contradictory results have been reported about the effectiveness of B supplementation alone and application time on yield, which seems more related to weather conditions, as cold and wet weather is more favorable for nut production or influencing variable such as training system (multistem, oak, among others) and trees density. Furthermore, the reports on Zn effects on hazelnut yield are scarce. Keshavarz et al. (2011) sprayed Persian walnut trees three times with 0, 1,050, and 1,750 mg L^−1^ Zn from September to May, obtaining higher nut yield at 1,050 mg L^−1^ Zn, whereas its effectiveness was enhanced when Zn was accompanied with B (174 mg L^−1^), as in our experiment. The variable Program in our experiment had an influence principally on Ca in leaves and more discreetly but still significantly in the kernel. However, as shown in PCA, kernel Ca has a great relation with kernel yield (%) (Figure 8A). Zinc foliar application (0.2% zinc sulfate) on mandarin increased several leaf nutrients, including Ca, similar to our results (Razzaq et al., 2013). However, B and Zn fertilizers did not affect the mineral composition of pomegranate fruits (Davarpanah et al., 2016).

According to our results, B and Zn application at the end of springtime in hazelnut planted in temperate areas such as Southern Chile can stimulate plant productivity (P3 and P1), whereas early spraying, such as P2, showed irregularity in stabilized nut production (Figure 4). The latter indicates that for this plantation area, October is a period in which the reproductive structures of the tree do not begin their development. Because in June/July (winter) is pollination time with hazelnuts showing an extended dichogamy by three or four months (Mehlenbacher, 1991; Beyhan and Marangoz, 2007). The B and Zn interaction was found as a strong correlation in the leaves for P1 (*r* = 0.89), P2 (*r* = 0.97), and P3 (*r* = 0.95) (Figure 6), which revealed that these micronutrients are interestingly related to each other in a combined application. This was also shown on PCA, where foliar B and Zn are highly correlated and influence mostly the treatments with high application rates of these elements (Figure 8A). A direct positive correlation was found between leaf Zn concentration and B (*r* = 0.77) and Zn concentrations (*r* = 0.60) in the kernels (Figure 6), demonstrating the importance of combined application of these micronutrients on nut species as reported by Brown and Shelp (1997) for walnut. Khan et al. (2011) reported not only significant increases of B and Zn in the leaves of Feutrell's early (*Citrus reticulata*) but also in the quality fruit parameters when the trees were sprayed with both micronutrients at a premature stage. Application of both B and Zn should be carried out under partialized program either during the whole productive season (P1) or to the end of springtime (P3) when most fruit sets occur. In general, a clear difference was observed between the three programs, and this seemed to depend on the distribution and prioritization of nutrients depending on the phenological phase.

Marketable nuts are highly appreciated by hazelnut agroindustry, and prices paid to growers depend on nut and kernel characteristics as industrial yield requirements. Some authors such as Solar and Stampar (2011) highlighted TDG as one of the cultivars with the highest kernel yield (%), after a phenological characterization of 16 hazelnut cultivars planted in Slovenia. Even though there was no significant interaction between PxT, B and Zn had a positive effect on kernel yield (%), particularly at a high rate (B_1600_+Zn_800_), where trees responded to sprayed treatments. Contrarily, Silva et al. (2003) found significant interaction among B rate, date of application, and harvest year of nuts, which could involve several factors that affect nut mass. Those authors reported that blank nuts amount was unaffected by B spraying, attributing this phenomenon to an annual variation (climatic or agronomic management). An interesting aspect is to study the effect of B and Zn on the shell morphology. However, in this sense, a thicker pericarp could be responsible for a reduced kernel yield (%). In fact, this could be the case because, as shown in PCA, lower rates of B and Zn are more related to the shell weight in P2 and P3 spaying programs (Figure 8A).

Both B and Zn have important roles in ameliorating reactive oxygen species (ROS) accumulation in plant tissues (Broadley et al., 2007; Marschner, 2012; Landi et al., 2013). As an essential micronutrient, zinc has broad functions in trees, from structural and catalytic to transcriptional regulation (Broadley et al., 2007; Marschner 2012). Reduced LP levels and hydrogen peroxide concentration were observed in soybean following Zn application, confirming its important role in mitigating oxidative stress under unfavorable conditions (Weisany et al., 2012). Boron is required for the stabilization of biomembranes, and it may protect them from ROS-induced damage (Cakmak and Römheld, 1997). Saadati et al. (2013) reported an increased phenolic content after the foliar application of B and Zn on olive trees during fruit ripening for two productive seasons. In both leaf and kernel, leaf B and Zn were negatively correlated with LP in all programs, which highlights the importance of both elements in decreasing damage of biomembranes in the plant tissues. However, the impact of B and Zn concentrations and timing on TPC seems to be not so clear. Increased DPPH activity especially in P3, in both B+Zn treatments, was not accompanied by TPE accumulation, indicating that other compounds with antioxidative properties (e.g., ascorbate) could be stimulated by B and Zn application. Further research will help to elucidate which B- ad Zn-inducible compounds contribute to improving the quality of nuts. In general, all programs induced changes in relation to antioxidative metabolism of leaves and kernels; however, these effects were more evident in P3 followed by P1 and the least in P2 spraying scenario.

## Conclusions

There is little knowledge concerning B and Zn interactions on hazelnut traits planted in temperate areas such as Southern Chile. Therefore, we performed comparative analyses of B and Zn dosage and four phenological stages of their foliar application (August–March in the Austral Hemisphere). We showed that these micronutrients could be partially sprayed at the end of spring in hazelnut, enhancing the management efficiency by reducing the application frequency, water volume, and applied rates. The results confirmed that in leaves, B and Zn were greatly increased, showing higher levels than those reported by the literature in other worldwide plantation areas, although no toxicity symptoms were detected. However, only Zn was significantly increased in kernels, increasing its nutritional value as recommended by the daily amount for human consumption. The treatments differentially affected the mineral status in leaves and kernels, with increased Ca and Na concentrations in the leaves. Increased RSA and reduced level of LP was observed in B+Zn-treated leaves and kernels in P2 and P3, respectively, but were not positively associated with B or Zn in tissues. The foliar supplementation with B and Zn had better efficiency at 800 and 400 mg L^−1^, respectively, under programs of four (P1) or two (P3) spraying applications, on the nut yield per plant or per hectare. From the agro-environmental point of view, two applications will result in a better productive strategy impacting agro-economical management during the growing season. Hazelnut has shown a great potential to produce nutritionally well-balanced and safe food in the coming years to improve commercial opportunities for growers in Southern Chile. In both leaves and kernels, the antioxidative capacity increased after B and Zn spraying (especially, at B_800_+Zn_400_), which may not be related to phenolic compounds.

Overall, this study contributes to understanding the effects of B and Zn spraying application in relation to phenological hazelnut phase. Knowing the correct timing and nutrient dosage is a prerequisite for obtaining the best plant performance and fruit qualitative features for future sustainable hazelnut production.

## Data Availability Statement

The raw data supporting the conclusions of this article will be made available by the authors, without undue reservation.

## Author Contributions

CM-G, DA, AL-E, and KO designed the research. CM-G, AL-E, and DA supervised the study. CM-G, AL-E, and FM analyzed the data. CM-G, AL-E, MR-D, FM, GO, and KO wrote the manuscript. All authors critically revised the manuscript and approved the final version.

## Conflict of Interest

The authors declare that the research was conducted in the absence of any commercial or financial relationships that could be construed as a potential conflict of interest.
